# LFA-1/ ICAM-1 promotes NK cell cytotoxicity associated with the pathogenesis of ocular toxoplasmosis in murine model

**DOI:** 10.1371/journal.pntd.0010848

**Published:** 2022-10-07

**Authors:** Nannan Gao, Chong Wang, Yiran Yu, Linding Xie, Yien Xing, Yuan Zhang, Yanling Wang, Jianjun Wu, Yihong Cai

**Affiliations:** 1 Department of Health Inspection and Quarantine, School of Public Health, Anhui Medical University, Hefei, China; 2 Department of Microbiology and Parasitology, the Provincial Laboratory of Pathogen Biology of Anhui, and the Key Laboratory of Zoonoses of Anhui, Anhui Medical University, Hefei, China; 3 Anhui Provincial Center for Disease Control and Prevention, Hefei, China; Universidad del Quindío: Universidad del Quindio, COLOMBIA

## Abstract

Ocular toxoplasmosis (OT) is one of the most common causes of posterior uveitis. However, the pathogenic mechanisms of OT have not been well elucidated. Here, we used C57BL/6 (B6) mice to establish OT by peroral infection with 20 cysts of the TgCtWh6 strain, and severe ocular damage was observed by histopathological analysis in the eyes of infected mice. RNA-sequencing results showed that infection with *T*. *gondii* increased the expression of the NK-mediated cytotoxicity gene pathway at Day 30 after ocular *T*. *gondii* infection. Both NK-cell and CD49a^+^ NK-cell subsets are increased in ocular tissues, and the expression levels of LFA-1 in NK cells and ICAM-1 in the OT murine model were upregulated upon infection. Furthermore, inhibition of the interaction between LFA-1 and ICAM-1 with lifitegrast, a novel small molecule integrin antagonist, inhibited the protein expression of LFA-1 and ICAM-1 in murine OT and NK cells, improved the pathology of murine OT and influenced the secretion of cytokines in the OT murine model. In conclusion, the interaction between LFA-1 and ICAM-1 plays a role in the early regulation of the CD49a^+^ NK-cell proportion in an OT murine model. LFA-1/ ICAM-1 may be a key molecule in the pathogenesis of OT, and may provide new insights for potential immunotherapy.

## Author summary

Ocular toxoplasmosis (OT), despite its high prevalence and impact worldwide, is currently understudied. An immunological approach to the disease is relevant given the particular immunological milieu where it happens (the eye) and the known importance of innate immunity in the resolution of the disease. We revealed that LFA-1/ICAM-1 is essential for inflammatory NK cells at the ocular region with TgCtwh6, which is the predominant clonal lineage (ToxoDB#9) in China. A higher percentage of CD49a^+^ NK1.1^+^ CD3^-^ cells and upregulated LFA-1/ICAM-1 expression in the OT murine model promoted inflammation and aggravated damage in OT. Moreover, lifitegrast is a novel small molecule integrin antagonist that blocks the binding of ICAM-1 to LFA-1. The amelioration of pathology and the diminished expression of inflammatory cytokines upon lifitegrast treatment could pave the way for more specific intervention in patients.

## Introduction

Ocular toxoplasmosis (OT) is caused by intraocular infection with *Toxoplasma gondii* (*T*. *gondii*), which is an obligate intracellular protozoan parasite that can affect all warm-blooded vertebrates, including humans [1]. OT is a major cause of infectious posterior uveitis worldwide and often leads to impairment of vision in the affected eye [2]. Current research on the genotypes of Toxoplasma virulence and pathogenicity has focused on three clonal lineages (I, II and III) from North America and Europe, where the type II strain shows the highest frequency, but the largest multifocal and haplotype analysis found that Toxoplasma species existed as six major clades with a biphasic geographical pattern distribution that corresponds to a few highly clonal genotypes that predominate in the northern hemisphere, whereas in South America, the parasite populations are characterized by a greater genetic diversity [3]. Additionally, the predominant clonal lineage in East Asia, particularly in China, has been reported as the Chinese 1 genotype (ToxoDB#9) [4, 5]. Both the severity of an episode and the chances of recurrence are related to the serotype of the parasite and the conditioned cytokine response of the host [6]. Patients infected by atypical strains have a specific intraocular cytokine pattern and retinochoroiditis toxoplasmosis susceptibility is linked to polymorphisms in cytokine genes [7, 8].

Activation and recruitment of inflammatory cells are key processes in the pathogenesis of OT [9]. Acute and chronic *T*. *gondii* infections selectively recruit natural killer (NK) cells and stimulate NK-cell activity [10]. Leukocyte function-associated antigen-1 (LFA-1) (α L β 2 or CD11a-CD18) is expressed on the surface of many immunocytes, including T cells, neutrophils, macrophages, and NK cells [11]. In addition to mediating firm adhesion to target cells, LFA-1–mediated signals inducing granule polarization are essential for NK-cell cytotoxicity and constitute an early signal for NK-cell activation [12–15]. Intercellular adhesion molecule-1 (ICAM-1, CD54), a cell surface glycoprotein, is expressed at low levels on endothelial cells and epithelial cells. It mediates not only the adhesion of leukocytes to the vascular endothelium but also the migration of leukocytes [16].

The discovery of ICAM-1 as a ligand for LFA-1 established a receptor–ligand pair for the key adhesion pathway and revealed that upregulation of ligand expression by inflammatory cytokines acts as an important switch for initiating adhesion. A low basal level of ICAM-1 expression was upregulated transcriptionally by cytokines (TNF-α, IL-1,IFN-γ) [17]. The LFA-1 interaction with ICAM-1 plays an essential role in the regulation of a variety of immunologic and inflammatory cell responses. Overall, the signal transduction mediated by LFA-1 and ICAM-1 is involved in the migration, cell adhesion, and activation processes of lymphocytes, as well as the formation of immune synapses, which makes LFA-1 and ICAM-1 closely associated with infection and autoimmune diseases [18, 19].

The upregulation of ICAM-1 expression in both human retinal pigment epithelial (RPE) cells and retinal vascular endothelial (RVE) cells has been reported after *T*. *gondii* infection [20]. Migration of *T*. *gondii* tachyzoites and *T*. *gondii*–infected dendritic cells (DC) across the retinal endothelium assisted by ICAM-1 *in vitro* [21, 22].However, the role of ICAM-1 and LFA-1 in the inflammatory response during OT has not been investigated.

This study defines the function of LFA-1 and ICAM-1 in sustaining inflammatory NK cells during intracellular pathogen infection. LFA-1/ICAM-1 can serve as a key molecule in the pathogenesis of OT and a potential novel therapeutic target for OT.

## Materials and methods

### Ethics statement

All animal experiments were approved by the Animal Experimentation Ethics Committee of Anhui Medical University (permit number 20211187). This study was conducted in agreement with the recommendations of the Guide for the Use and Care of Laboratory Animal of Anhui Medical University.

### Mice and parasites

175 C57BL/6 female mice at 6–8 weeks of age, weighing about 12–18 g, were purchased from the Experiment Animal Center of Anhui Medical University (Anhui, China). All mice had normal findings on physical and ophthalmic examinations.

The cysts of an avirulent Chinese 1 strain (ToxoDN#9) ——TgCtwh6[4, 5], were prepared from the brains of Kunming (KM, outbred) female mice perorally infected 2 months before. The brain was homogenized in 2 mL of sterile saline and cysts numbers determined microscopically in 20 μL of suspension.

### Lifitegrast

Lifitegrast and sodium carboxymethyl cellulose were purchased from Med Chem Express. 0.5% sodium carboxymethyl cellulose served as the vehicle. Then, lifitegrast (1%) was prepared by adding 1 ml of 0.5% sodium carboxymethylcellulose per 0.01 g.

### Experimental schedule

To induce OT, the mice were infected perorally with 20 cysts of *T*. *gondii* from Day 0 [23, 24]. Uninfected, normal mice were used as controls. In a second experiment, uninfected mice served as the negative control, and infected mice treated with vehicle from Day 0 served as ‘Vehicle 1’ or Day 14 served as ‘Vehicle 2’. Infected mice treated with lifitegrast from Day 0 as ‘Lifitegrast 1’ or Day 14 as ‘Lifitegrast 2’. A single drop (5 μl) was administered to both eyes thrice daily. All mice were killed by CO_2_ inhalation at Day 30 post infection, and the eyes were enucleated.

### Histopathology

At 30 days post-infection (dpi), mice were sacrificed by CO_2_ asphyxiation. Their eyes were isolated, directly fixed in 4% buffered formaldehyde (Guangzhou Chemical Reagent Factory, China) and embedded in paraffin. Then, each piece was serially cut into several sections (5 μm thick) and stained with hematoxylin and eosin (H&E). Images were obtained on a light microscope (Leica, Germany) equipped with a charge-coupled device camera.

### Detection of *T*. *gondii* by PCR

DNA extraction from the retina of each mouse was performed using a DNA Extraction Kit (TaKaRa, Japan) in accordance with the manufacturer’s protocol. PCR amplification of the ITS-1 gene [25] was performed by using the primers ITS-1 5′-GATTTGCATTCAAGAAGCGTGATAGTAT-3′ and ITS-1 reverse 5′-AGTTTAGGAAGCAATCTGAAAGCACATC-3′.The PCR mix consisted of 12.5 μl of PCR Master Mix 1× (Promega, USA), 1 μl of each primer at 1 μM, 2.5 μl of 1× PCR buffer and 8 μl of DNA sample in a final volume of 25 μl. The first step of amplification was 3 min of denaturation at 95°C. This step was followed by 35 cycles, with 1 cycle consisting of 30 s at 55°C at the annealing temperature, and 30 s at 72°C. All PCRs were performed in a thermal cycler (Biometra, Germany). The amplified cDNAs were electrophoretically separated on 1% agarose gels containing ethidium bromide and recorded using a digital gel documentation system (Bio-Rad, USA).

### Immunohistochemical analysis

Briefly, sections were dewaxed and hydrated, and then antigen sites were unmasked using citrate buffer (0.01 M, pH 6.0) at 100°C for 30 min and quenched with 3% H_2_O_2_ in Tris-buffered saline (pH 7.6) to inhibit endogenous peroxidase activity. The slides were incubated with avidin/biotin blocking solution (Vector Laboratories, Burlingame, CA, USA). Subsequently, sections were incubated with rabbit anti-IFN-γ, TNF-α, and IL-10 (1:500, Proteintech, Wuhan, China) overnight at 4°C, with a corresponding Alexa 488- or 546-conjugated secondary Ab (Invitrogen) for 45 min, and finally with Hoechst 33342 stain for 1 min. Images were taken under a light microscope (Leica, Germany). Image J version 1.8.0 software was used to assess the area and the integrated optical density (IOD) value of the positive dyed region. Average optical density = IOD/Area. The optical density of the tissue areas from five randomly selected fields (magnification, 400 ×) were counted in a blinded manner and subjected to statistical analysis.

### mRNA library construction and sequencing

Total RNA from the whole eye was isolated and purified using TRIzol reagent (Accurat Biology) following the manufacturer’s procedure. The RNA amount and purity of each sample was quantified using NanoDrop ND-1000 (NanoDrop, Wilmington, DE, USA). The RNA integrity was assessed by Bioanalyzer 2100 (Agilent, CA, USA) with RIN number >7.0, and confirmed by electrophoresis with denaturing agarose gel. Poly (A) RNA is purified from 1μg total RNA using Dynabeads Oligo (dT)25-61005 (Thermo Fisher, CA, USA) using two rounds of purification. Then the poly(A) RNA was fragmented into small pieces using Magnesium RNA Fragmentation Module (NEB, cat. e6150, USA) under 94°C 5-7min. Then the cleaved RNA fragments were reverse-transcribed to create the cDNA by SuperScript II Reverse Transcriptase (Invitrogen, cat. 1896649, USA), which were next used to synthesis U-labeled second-stranded DNAs with E. coli DNA polymerase I (NEB, cat.m0209, USA), RNase H (NEB, cat.m0297, USA) and dUTP Solution (Thermo Fisher, cat. R0133, USA). An A-base is then added to the blunt ends of each strand, preparing them for ligation to the indexed adapters. Each adapter contains a T-base overhang for ligating the adapter to the A-tailed fragmented DNA. Single- or dual-index adapters are ligated to the fragments, and size selection was performed with AMPureXP beads. After the heat-labile UDG enzyme (NEB, cat.m0280, USA) treatment of the U-labeled second-stranded DNAs, the ligated products are amplified with PCR by the following conditions: initial denaturation at 95°C for 3 min; 8 cycles of denaturation at 98°C for 15 sec, annealing at 60°C for 15 sec, and extension at 72°C for 30 sec; and then final extension at 72°C for 5 min. The average insert size for the final cDNA library was 300±50 bp. At last, we performed the 2×150 bp paired-end sequencing (PE150) on an Illumina Novaseq 6000 (LC-Bio Technology CO, Ltd, Hangzhou, China) following the vendor’s recommended protocol.

### Quantitative real-time PCR

Total RNA was extracted from the whole eye using an RNA Extraction Kit (TaKaRa, Japan) according to the manufacturer’s protocol. The absorbance values at 260 and 280 nm were used to estimate total RNA purity (NanoDrop, Wilmington, DE, United States). For cDNA synthesis, a cDNA Synthesis Kit (TaKaRa, Japan.) was used; the total amount of RNA used in quantitative real-time PCR (qRT–PCR) for each sample was 1 μg. Next, qRT–PCR was performed using SYBR Green qPCR Master Mix (TOYOBO, Japan) on a capillary-based LightCycler instrument (Roche) following the manufacturer’s instructions. The following primers were used in our study: IFN-γ forward, 5′-ATAAGCGTCATTGAATCACACC;IFN-γ reverse, 5′-TGGCAATACTCATGAATGCATC-3′;TNF-α forward, 5′-CAGGCGGTGCCTATGTCTC-3′; TNF-α reverse, 5′-CGATCACCCCGAAGTTCAGTAG-3′; ICAM-1 forward, 5′-GTGTGCCATGCCTTTAGCTC-3′; ICAM-1 reverse, 5′-CTGATCTTTCTCTGGCGGTT-3′; ITGAL forward, 5′-GTCTCGGACATGCGATCAGAA-3′; ITGAL reverse, 5′-GGCGGGACGATTTTGTAACAT-3′; ITGB2 forward, 5′-AGCAGAAGGACGGAAGGAACATTTAC3′; ITGB2 reverse, 5′-ATGACCAGGAGGAGGACACCAATC-3′. Relative mRNA expressions of each target gene were normalized to the housekeeping gene GAPDH mRNA, and the results are expressed as the fold change compared to uninfected controls.

### Western blot analysis

Western blot analysis was performed using routine protocols. Briefly, protein extracts (50 μg) from the whole eye were separated in 10% SDS-polyacrylamide gels and then transferred onto nitrocellulose membranes. Rabbit polyclonal antibodies against ICAM-1, IFN-γ, IL-10, cleaved-caspase3 (Wanleibio, China), CD11a, CD18, and TNF-α (Affinity Bioscience, USA) were incubated with the membranes overnight at 4°C. The membranes were washed in TBST (20 mM Tris-HCl pH 7.5, 150 mM NaCl, 0.1% Tween 20) 3 times, incubated with the indicated HRP-conjugated goat anti-rabbit IgG (Proteintech) antibody for 1 h at room temperature and then washed in TBST 3 times. A Super Signal Chemiluminescent detection system (Bio-Rad) was used to detect the signals.

### Flow cytometry

The eyes were collected from the mice as reported previously [26]. A single cell suspension was prepared by digestion for 30 min at 37°C with collagenase (1mg/ml) and DNAse (100 ug/ml) in RPMI-1640. Spleen samples were subjected to hypotonic red blood cell lysis. Single-cell suspensions were obtained from eyes and spleen by macerating the tissues through 70-m nylon mesh filters (BD Falcon, Bedford, MA).

The single-cell suspensions from eyes and spleen were washed with FACS buffer (PBS plus 2% FBS plus 0.1% Sodium Azide) followed by blocking of the Fc (CD16/32) receptors with anti-CD16/32 (clone 2.4G2) antibody prior to cell surface staining. To stain cell surface antigens, single cell suspensions were incubated at 4°C for 30 min in the 100 ul volume of FACS buffer containing fluorochrome-conjugated antibodies. The fluorochrome conjugated antibodies used for cell surface staining were PE-Cy7-NK1.1, APC-R700-CD3, APC-CD49a, PE-LFA-1(BioLegend, USA).

### Statistical analysis

Data were analyzed by SPSS16.0 software and were expressed as mean ± SEM. All graphs were performed using GraphPad Prism 5 software. Two-tailed Student’s t-test or Mann Whitney U test was applied to determine the statistical significance between two groups. ANOVA tests were applied for the multiple sets of data. The results were considered statistically significant at P<0.05.

## Results

### Establishment of the OT murine model by TgCtwh6

**Fig 1:**
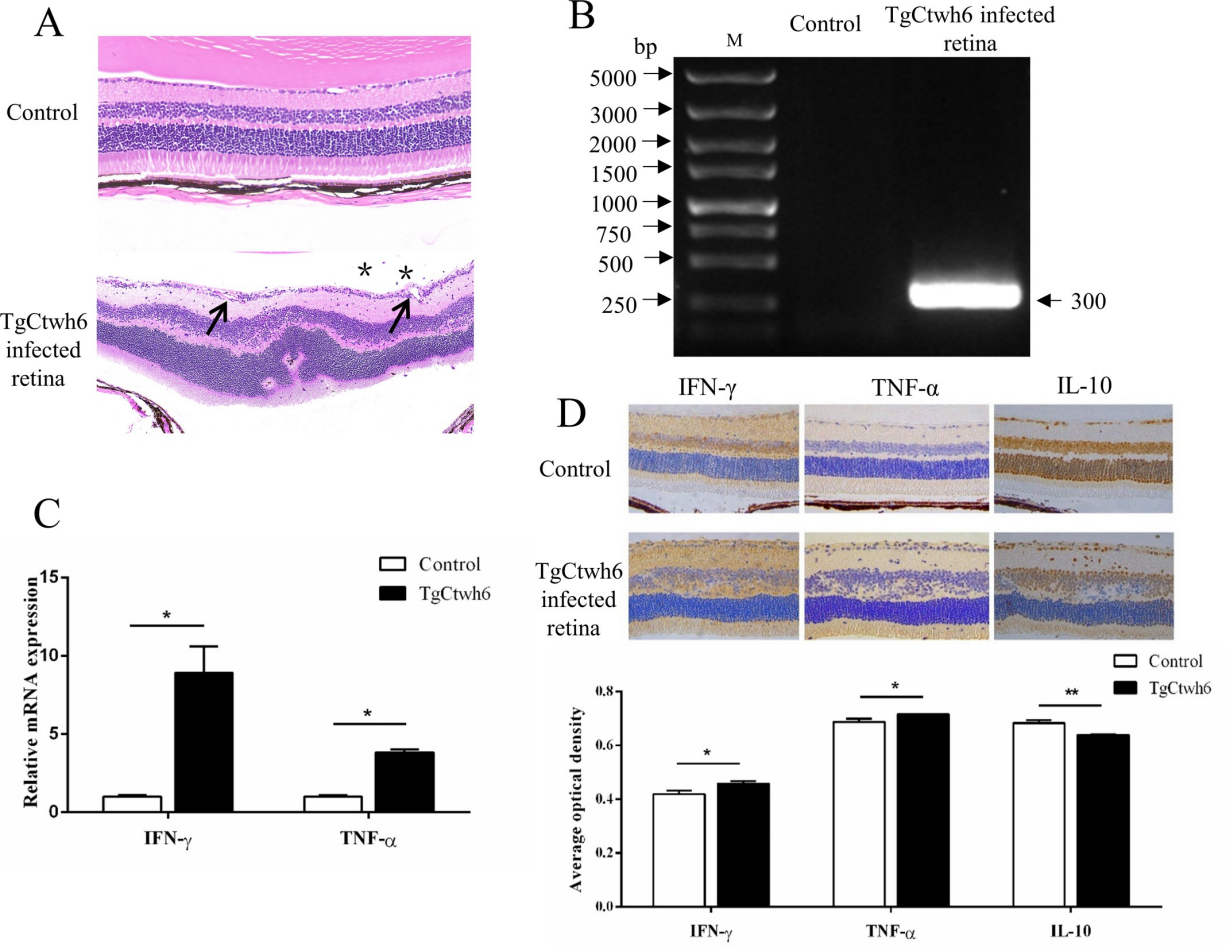
Histopathological changes in ocular tissues infected with the TgCtwh6 strain. Uninfected mice; infected mice at Day30. (A)Retina of a control eye from normal non-infected mice showing the normal architecture. All layers are present and unaltered. General aspect of TgCtwh6 infected retina showing the alteration of the retinal layer. Inflammatory cells were seen in the vitreous (_*_). Retinal vasculitis (black arrowhead) was evident and disorganization of the retinal architecture. Original magnification 400 ×, H&E stain. (B)Toxoplasma-ITS-1 PCR results, M: DNA Marker, n = 10 mice. (C)The mRNA expressions of IFN-γ and TNF-α in ocular tissues of naive mice and *T*. *gondii*-infected mice were measured by using a Real-time quantitative PCR. GAPDH was used as an internal control. The data were analyzed using Mann Whitney U test. mean ± SEM, *p < 0.05. (D)Representative experimental retinas stained for IFN-γ, TNF-α and IL-10 from control and OT murine model by immunohistochemistry as described in the Methods. 400 ×. Quantification of IFN-γ, TNF-α and IL-10 expression levels in retinas by the software Image J. Values represent the mean ± SEM, *P<0.05, **P<0.01.

Infected eyes were enucleated from mice at 30 days after parasite challenge, and histopathological changes were analyzed. Compared with that of the control eye, a large number of infiltrating lymphocytes were found within the vitreous, retinal vasculitis was evident, the retinas were edematous, and a disorganization of retinal architecture tissue was observed in the infected eye of the OT groups (Fig 1A). We detected the *T*. *gondii-*specific gene (ITS-1) expression in the OT murine model [25]. All PCR tests in uninfected mice were negative, and samples from infected mice were positive (Fig 1B). Previous studies have reported that the inflammatory mediators IFN-γ, TNF-α and IL-10 play an important role in intraocular infection in OT [27–31].To examine their distribution within the retina, we studied their protein expression by immunohistochemistry. As shown in Fig 1C and 1D, both inTgCtwh6 infected whole eye and retina, there was a significant increase in IFN-γ and TNF-α expression, while the IL-10 expression level was significantly decreased in the retinas of *T*. *gondii*-infected mice.

### Infection with *T*. *gondii* increased the expression of the NK-mediated cytotoxicity gene pathway

**Fig 2:**
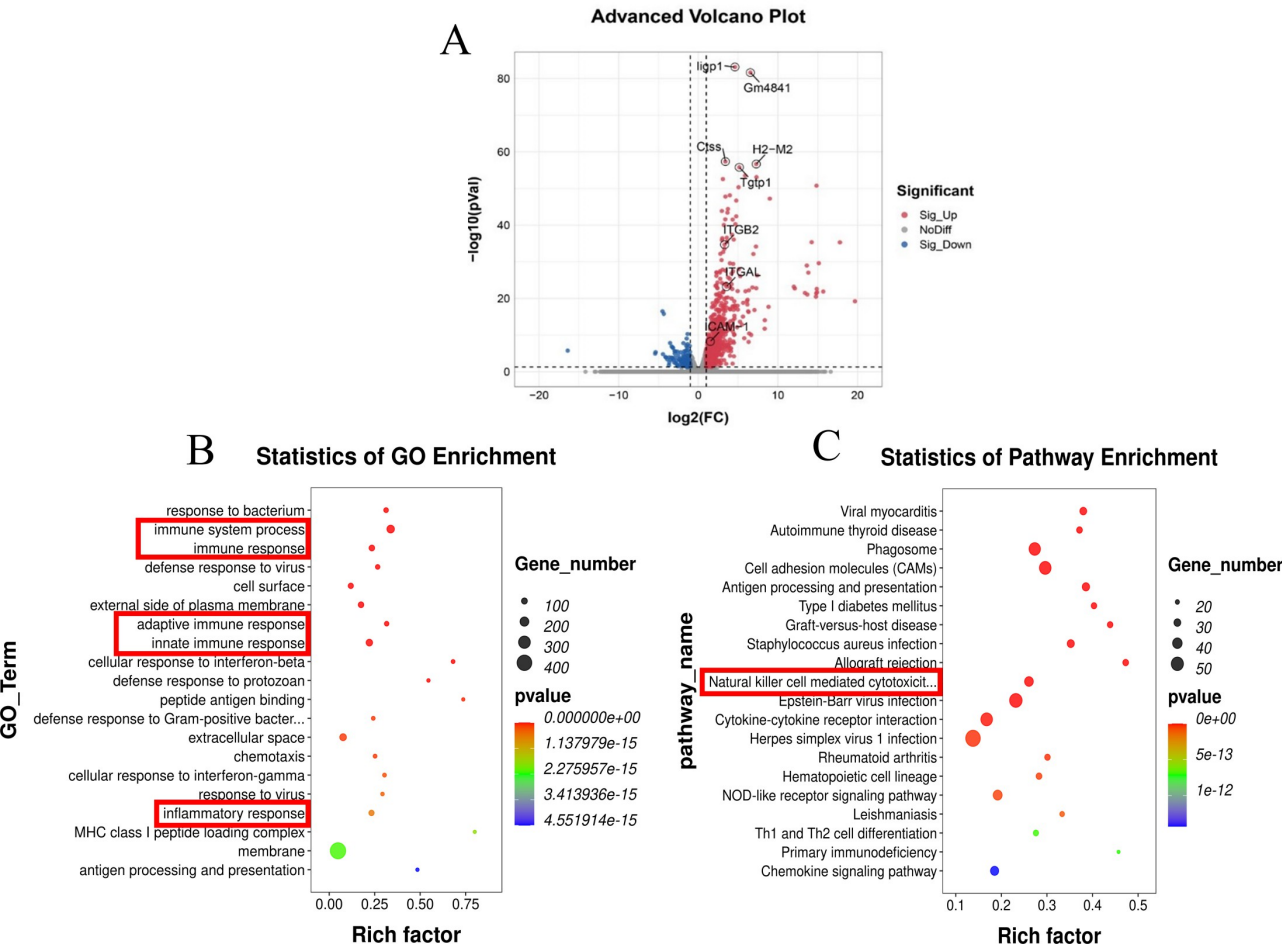
mRNA sequencing and regulatory gene analysis at Day 30 after TgCtwh6 infection. (A) Volcano plots showing Log2(Fold Change) differences versus–Log10(False Discovery Rate) for indicated comparisons, using pooled data from each indicated sample type. Genes exhibiting Log2FC > 1 and FDR < 0.1 are colored. Red represents up-regulated significantly differentially expressed genes, blue represents down-regulated significantly differentially expressed genes and grey dots represent non-significant differentially expressed genes. The differentially expressed mRNAs were selected with fold change > 2 or fold change < 0.5 and p value < 0.05 by R package edgeR or DESeq2, and then analysis GO enrichment (B) and KEGG enrichment (C) to the differentially expressed mRNAs.

Based on the RNA-sequencing data, we obtained 709 differentially expressed genes, with 601 upregulated genes and 108 downregulated genes (Fig 2A). Functional enrichment analyses of the differentially expressed genes offered a biological understanding of these genes. The top enriched Gene Ontology (GO) term in biological processes was the immune system process (Fig 2B). In the Kyoto Encyclopedia of Genes and Genomes (KEGG) pathway enrichment analysis, several enriched pathway terms and 31 differentially expressed genes were enriched in the NK-mediated cytotoxicity pathway (Fig 2C and S1 Table). These results suggested that *T*. *gondii*-induced infection may be associated with the NK-mediated cytotoxicity pathway in murine OT.

### Both NK1.1^+^ CD3^-^ cells and NK1.1^+^ CD3^-^ CD49a^+^ NK cells are increased in the eyes of the OT murine model

**Fig 3:**
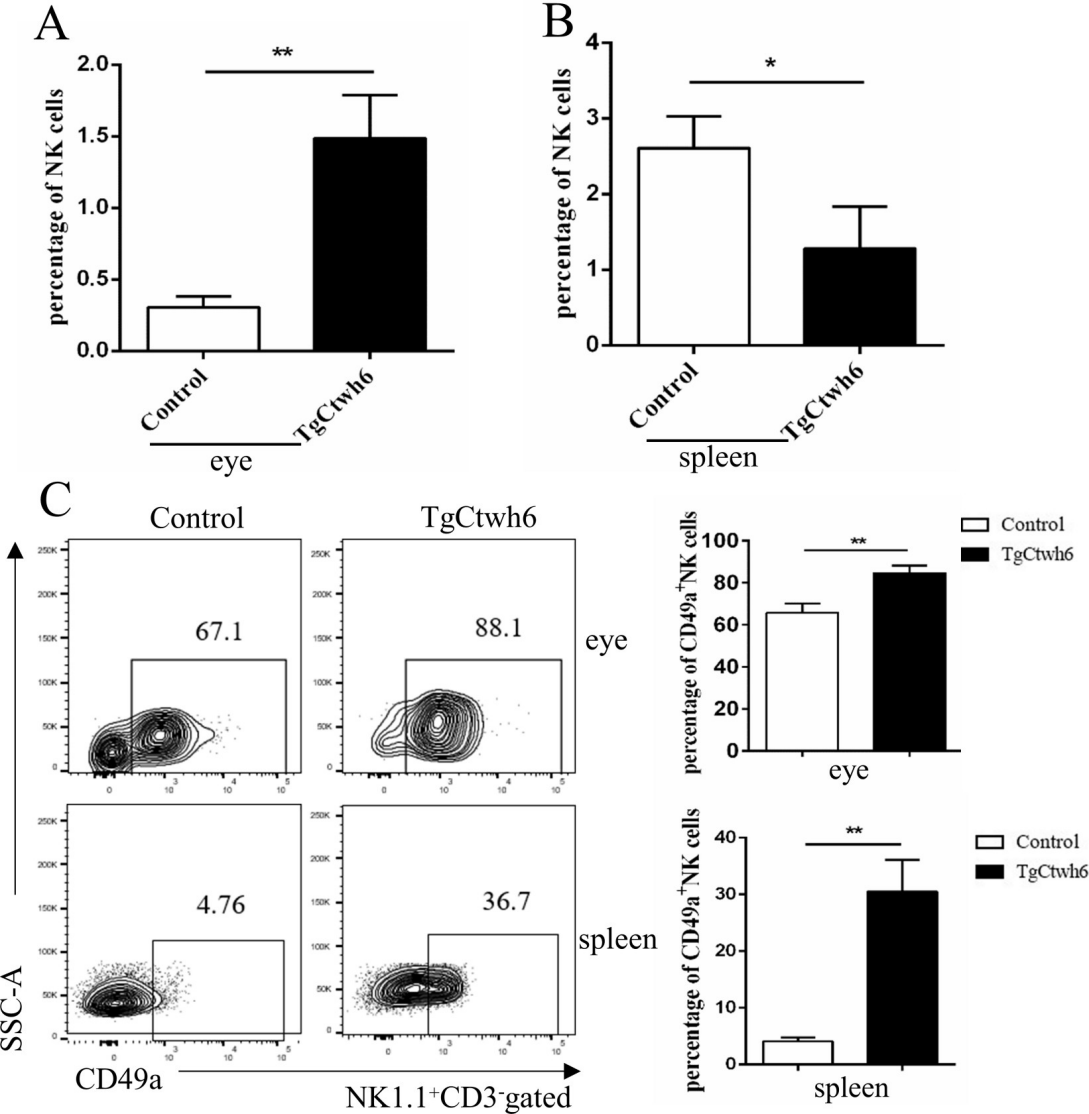
Representative flow cytometry plots for the analysis of NK cells and CD49a^+^ NK in the eyes and spleen of uninfected and Day 30-infected mice. Percentage of NK (CD3^-^ NK1.1^+^) cells in lymphocytes in the infected eyes (A) and spleen (B) from representative murine OT and control mice. (C) The percentage of CD49a^+^ NK (NK1.1^+^ CD3^-^ CD49a^+^) cells was increased in infected eyes and spleen in the OT murine model. Pools of 10 mice were used and experiments were replicated three times. Values represent the mean ± SEM, *P<0.05, **P<0.01.

According to the sequencing information, *T*. *gondii* promotes the expression of inflammatory factors and induces infection through the NK-mediated cytokine pathway. To confirm that the sequencing information was reliable, we detected the NK (CD3^−^ NK1.1^+^) cells percentage in OT murine model. CD3^−^ NK1.1^+^ cells were increased in infected eyes at Day 30 after infection (Fig 3A). The spleen is an important immune organ, and the percentage and phenotype of NK cells in the spleen were also analyzed. However, the percentage of CD3^−^ NK1.1^+^ cells from the infected spleen was significantly decreased compared with that of the control spleen (Fig 3B). CD49a, an integrin alpha subunit, can bind collagen and laminin and has been identified as a surface marker of tissue-resident NK (trNK) cells in mice [32].The percentage of CD49a^+^ NK cells was significantly increased in response to infection compared with that observed in the control eyes and spleen (Fig 3C).

### LFA-1 expression in NK cells and ICAM-1 in the eyes were upregulated in the OT murine model

**Fig 4:**
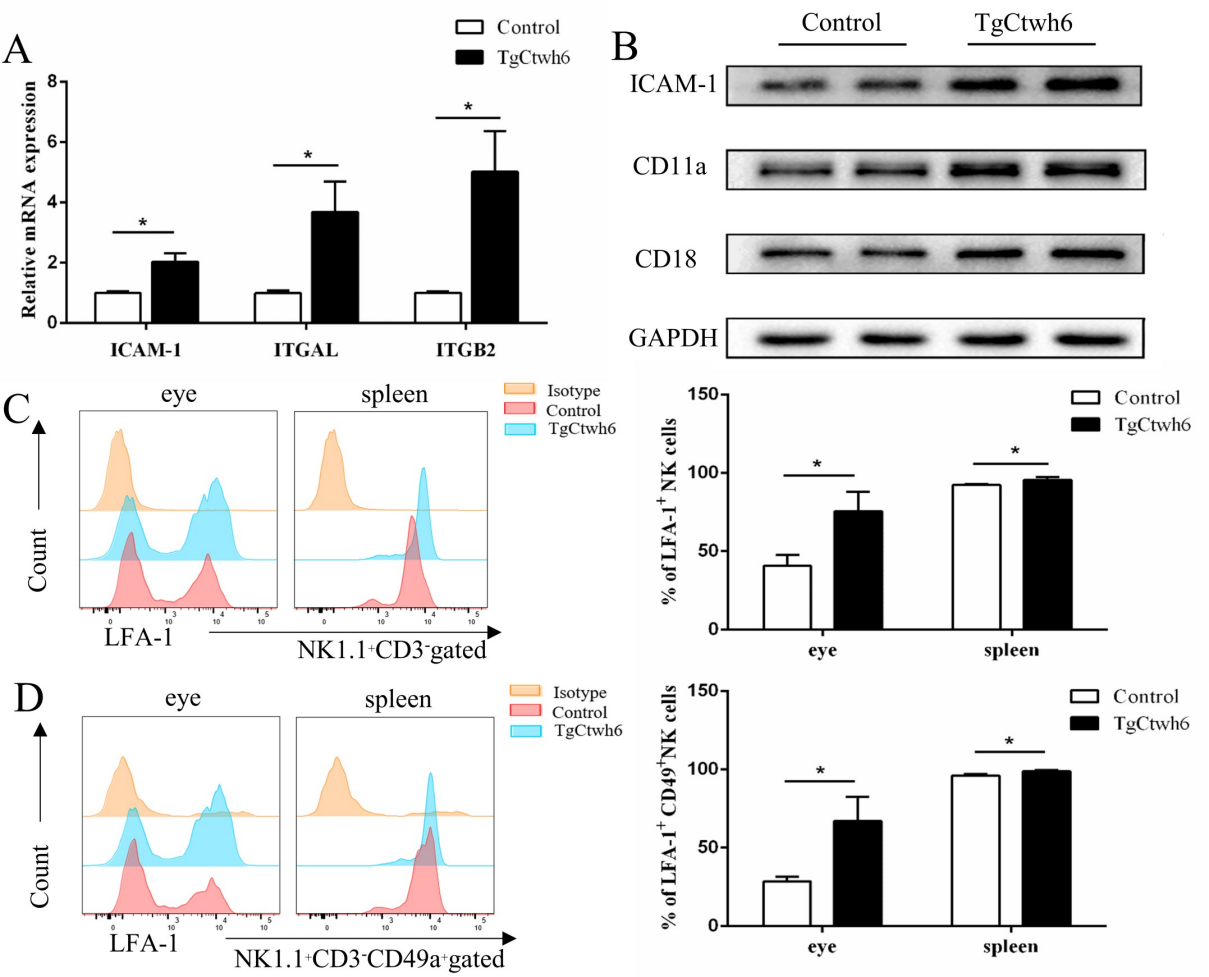
*T*. *gondii* infection upregulates the expression of ICAM-1 and LFA-1 by NK cells in murine ocular tissue. (A) The mRNA expressions of ICAM-1 and LFA-1 (ITGAL/ITGB2 gene) in ocular tissues of uninfected mice and TgCtwh6-infected mice were measured by using a Real-time quantitative PCR. GAPDH was used as an internal control. The results were representative of 3 independent experiments and the data were analyzed using Mann Whitney U test. mean ± SEM, *p < 0.05. (B) Western blot analysis showed that the protein expression of ICAM-1 and LFA-1 (CD11a/CD18) was upregulated in the eyes in comparison with the uninfected eye. GAPDH was used as an internal control. The results are from a pool of protein from two mice and are representative of results in three independent experiments. Expressions of LFA-1 on infiltrating CD3^−^NK1.1^+^ cells (C) and CD49a^+^ NK cells (D) in the eyes or spleen. Pools of 10 mice were used and experiments were replicated three times. The results were representative of 3 independent experiments and represented as mean ± SEM. The data were analyzed using Student’s t-test. mean ± SEM, *p < 0.05; **p < 0.01.

To investigate whether LFA-1 and ICAM-1 are key molecules in the pathogenesis of murine OT, we detected the expression of ICAM-1/LFA-1 in eyes and NK cells. LFA-1 is composed of α chain CD11a (ITGAL gene) and β chain CD18 (ITGB2 gene) dimers and belonging to the integrin family. ITGAL, the gene encoding CD11a, is located on chromosome 16p11.2 [33]. ITGB2 gene is situated on chromosome 21 and encoding CD18 [34]. Compared with naive mice, the mRNA expression levels of ICAM-1 (P <0.05), ITGAL (P < 0.05), and ITGB2 (P < 0.05) were significantly increased in the eyes of *T*. *gondii*-infected mice (Fig 4A). In addition, the protein expression levels of ICAM-1, CD11a and CD18 were significantly increased in OT murine model (Fig 4B). LFA-1 was expressed at higher levels on CD3^−^NK1.1^+^ cells (Fig 4C)and CD49a^+^ NK cells (Fig 4D) in infected eyes or the spleen compared to non-infected mice.

### Lifitegrast reduced the percentage of NK1.1^+^ CD3^-^ CD49a^+^ NK cells in the OT murine model

**Fig 5:**
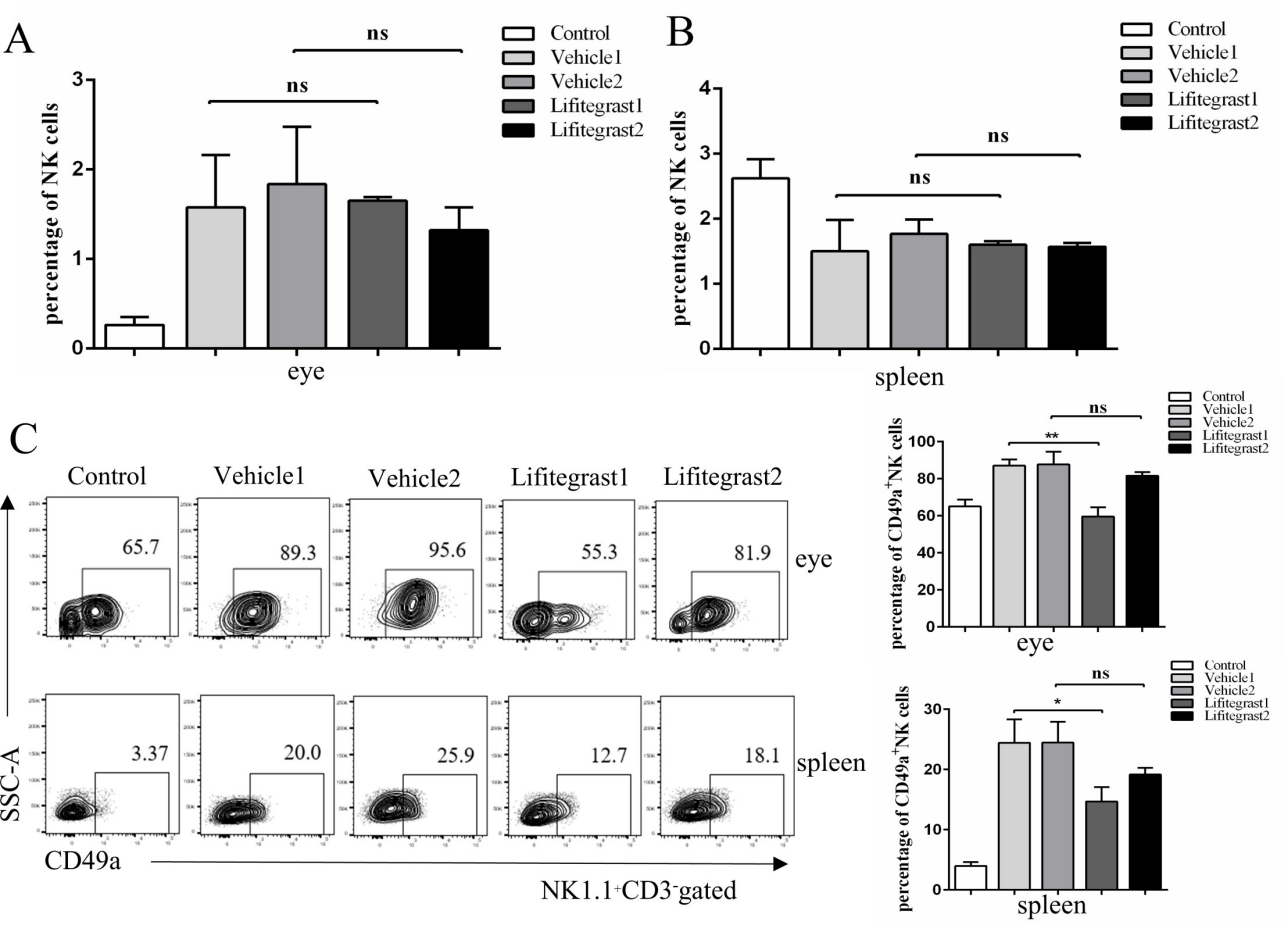
The percentages of CD3^-^ NK1.1^+^ cells and CD49a^+^ NK cells were assessed in the eyes and spleen at Day 30 after infection in the OT murine model treated with vehicle or lifitegrast. (A-B) Representative flow cytometry plots for the analysis of NK cells (CD3^-^ NK1.1^+^) and (C) CD49a^+^ NK cells. n = 5–11 mice; mean ± SEM; one-way ANOVA test; ns not significant, *p<0.05, **p<0.01. Data are representative of three independent experiments.

To determine whether LFA-1/ICAM-1 mediates NK-cell trafficking into the target organ, we examined the percentage of NK cells in the eyes and spleen after lifitegrast treatment. Lifitegrast is a novel small molecule integrin antagonist that blocks the binding of ICAM-1 to LFA-1 [35]. On Day 30 after infection, we harvested recipient eyes and assessed the CD3^-^ NK1.1^+^ cells percentage in the eyes and spleen by flow cytometry. Interestingly, lifitegrast did not reduce the increased percentage of CD3^-^ NK1.1^+^ cells in the eyes (Fig 5A) and spleen (Fig 5B).

As CD49a is an important marker of NK-cell subsets, we analyzed the effect of lifitegrast on the percentage of CD49a^+^ NK cells in infected eyes and spleen. Our results revealed that CD49a^+^ NK cells from infected eyes (P < 0.01) and the spleen (P < 0.05) were decreased in the Lifitegrast 1 group compared with the Vehicle 1 group but failed to influence the proportion of CD49a^+^ NK cells in the Lifitegrast 2 group. (Fig 5C).

### Lifitegrast treatment inhibited the protein expression of LFA-1 and ICAM-1 in murine OT and NK cells

**Fig 6:**
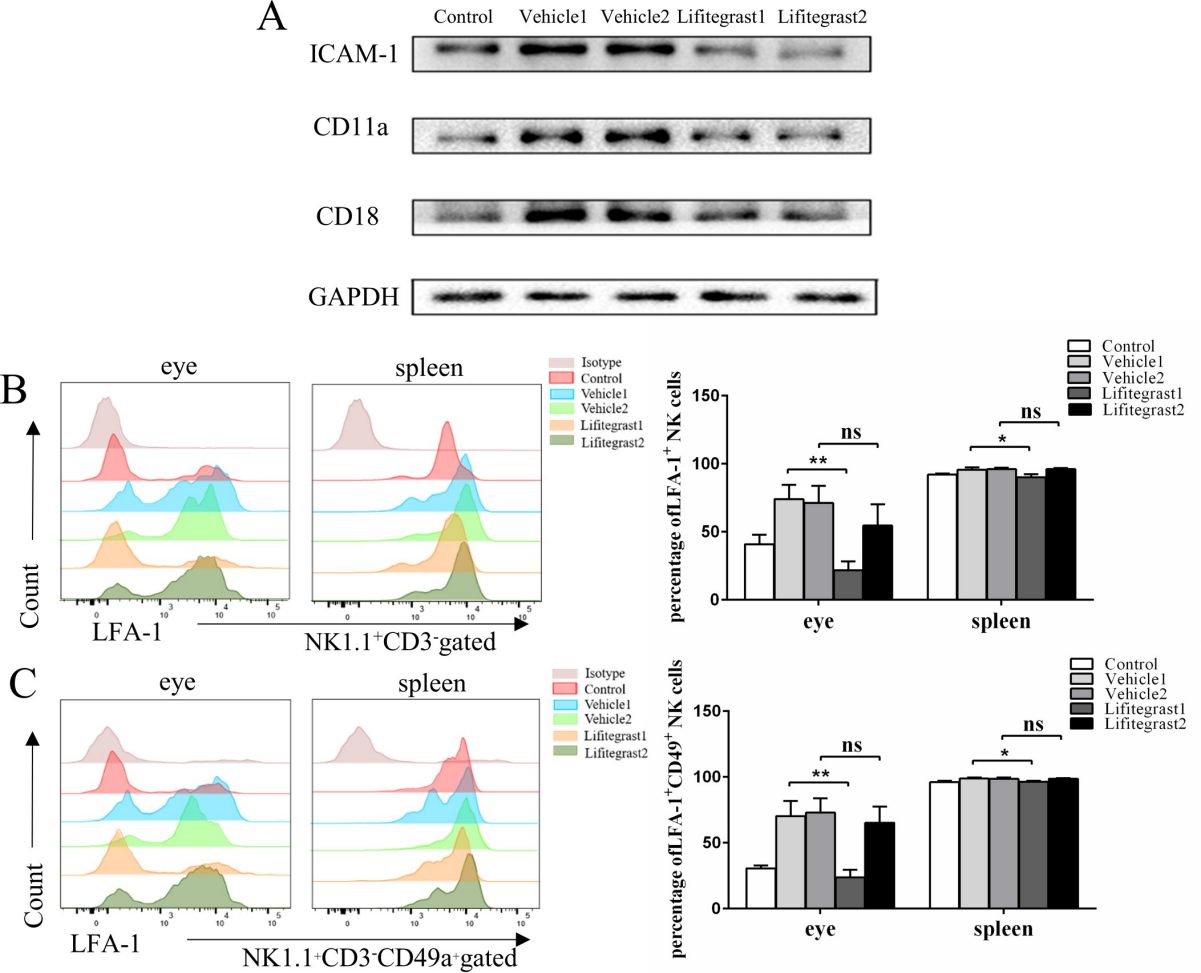
Topical lifitegrast administration inhibited the expression of LFA-1 and ICAM-1 in OT murine model. (A) Western blot analysis showed that ICAM-1 and LFA-1 were decreased in infected eyes after lifitegrast treatment. GAPDH was used as an internal control and the results are from a pool of protein from two mice. Flow cytometry analysis of LFA-1 expression in CD3^-^ NK1.1^+^ cells (B) and CD49a^+^ NK cells (C) from lifitegrast-treated eyes or spleen of OT murine model. Pools of 10 mice and the experiment was repeated three times. The differences from the groups were compared using an ANOVA test. ns, not significant; *p<0.05; **p<0.01.

Lifitegrast inhibits the binding of LFA-1 to ICAM-1. The signal transduction mediated by LFA-1 and ICAM-1 is involved in the adhesion, migration, and activation processes of lymphocytes [18],which may decrease the production of LFA-1 and ICAM-1 in the OT murine model. Therefore, we used western blot analysis to determine the expression of CD11a, CD18 and ICAM-1 in ocular tissues. Compared to the vehicle-treated group, the eyes of the lifitegrast-treated group showed a marked decrease in LFA-1 and ICAM-1 protein expression (Fig 6A). Our results show that lifitegrast treatment decreased the expression of LFA-1 on CD3^−^ NK1.1^+^ cells (Fig 6B) and CD49a^+^ NK cells (Fig 6C) in the Lifitegrast 1 group compared with that of Vehicle 1 group but failed to influence the expression of LFA-1 on CD3^−^ NK1.1^+^ cells and CD49a^+^ NK cells in the Lifitegrast 2 group.

### Lifitegrast treatment ameliorated the pathology of OT and influenced the secretion of cytokines

**Fig 7:**
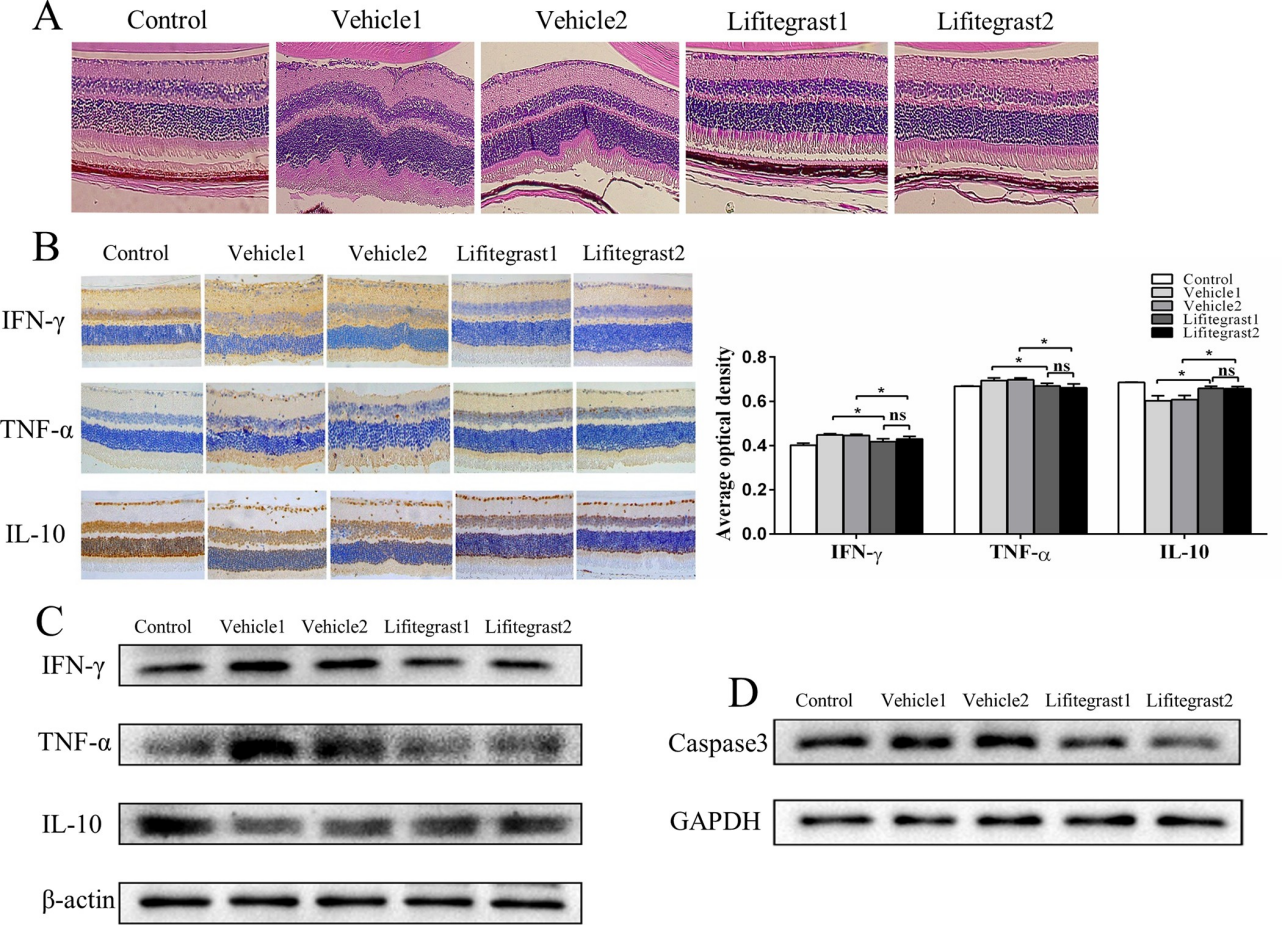
Topical lifitegrast administration reduced ocular inflammation. (A) Histology of the eye at Day 30 after infection in the OT murine model treated with vehicle or lifitegrast. Hematoxylin and eosin; 400 ×. (B) Representative experimental retinas stained for IFN-γ, TNF-α and IL-10 from vehicle or lifitegrast-treated murine OT. Immunohistochemistry showed that the production of pro-inflammatory cytokines (IFN-γ, TNF-α) but not anti-inflammatory cytokines (IL-10) was significantly inhibited by lifitegrast treatment. 400 ×. Quantification of IFN-γ, TNF-α and IL-10 expression levels in retinas by the software Image J. ns, not significant; *p<0.05. (C) Western blot analysis showed that IFN-γ and TNF-α were decreased, while IL-10 was increased in infected eyes after lifitegrast treatment. β-actin was used as an internal control. (D)Western blot analysis showed that caspase-3 were decreased in infected eyes after lifitegrast treatment. GAPDH was used as an internal control.

Histologic examination of the eyes at Day 30 after infection treated three times starting from Day 0 showed that recipient mice treated with vehicle exhibited damage with a disorganization of retinal architecture and infiltration in the retina, whereas those treated with lifitegrast had a well-preserved retinal structure, with much milder ocular infection. Most importantly, when treatment with lifitegrast was started from Day 14 (disease onset), it was equally effective (Fig 7A). As shown in Fig 7B and 7C, compared with vehicle treatment, lifitegrast treatment caused significant inhibition of the secretion of IFN-γ and TNF-α in the OT murine model. However, lifitegrast treatment increased the expression of IL-10 in the OT murine model. These results indicate that lifitegrast affects the secretion of cytokines in the OT murine model. Because activation of LFA-1 receptors has been implicated in preventing apoptosis, we examined caspase-3, a pro-apoptotic protein, expression following infection with or without lifitegrast treatment. Experimental eyes of vehicle-treated mice had higher expression of caspase-3 than those of lifitegrast-treated mice (Fig 7D).

## Discussion

The most common clinical manifestation of *T*. *gondii* infection is ocular retinochoroiditis, which in response to the organism causes intraocular infection and may result in a disorganization of retinal architecture tissue and permanent loss of vision. Despite the fact that OT is a potential blinding disease, there is no curative treatment for preserving vision and preventing recurrence thus far. NK cells are required for acute *T*. *gondii* control, regulate infection via IL-10, and may contribute to adaptive immune responses [36]. The spleen is an important immune organ, and the NK-cell numbers of mice that were infected with 15 cysts perorally and harvested 7 days after infection significantly increased in the spleen [10]. Some studies have reported increased production of NK cells in autoimmune uveitis [26, 37, 38], and it has been shown that NK cells can be used as a therapeutic target in experimental autoimmune uveoretinitis (EAU), which can be improved by depleting NK cells with antibodies [39]. However, alterations in the proportion of NK cells in OT murine model have not been reported. Therefore, we examined the percentage of NK cells in the eyes and spleen during the chronic phase of *T*. *gondii* infection and our results showed a significant increase in the percentage of NK cells in ocular tissue.

CD49a has been identified as atypical marker of tissue-resident NK (trNK) cells [32]. TrNKs were first identified in the murine liver and then rapidly expanded to various other tissues, such as the uterus, skin, kidney and adipose tissue [32, 40–42]. They may be involved in maintaining local homeostasis in tissues, participating in a variety of important pathophysiological processes, and may develop memory-like characteristics [43]. After 21 days mice were infected with 200 tachyzoites, and *T*. *gondii* infection induced the conversion of NK cells into ILC1-like (Eomes^-^CD49a^+^) cells in the spleen, which was a permanent change [44]. CD49a expression on NK cells is upregulated in tumors, and the accumulation of tumor-infiltrating CD49a^+^ NK cells is associated with poor prognosis for human hepatocellular carcinoma [45]. Moreover, CD49a^+^ NK cells produce high levels of proinflammatory cytokines, such as IFN-γ and TNF-α, and infection alters their expression in stimulated CD49a^+^ NK cells [46, 47]. We found a significant increase in the proportion of CD49a^+^ NK cells in ocular tissue during *T*. *gondii* infection, suggesting that this cell subset may be associated with the progression and prognosis of OT. Some studies have reported that Inc-49a is a positive regulator of CD49a in human decidual NK cells [48] and that γδT cells promote the activation of CD49a^+^ NK cells and upregulate their surface cytotoxic effector molecules (e.g., perforin) through CD137/CD137 L signaling in liver fibrosis [49]. Manipulation of CD49a cell changes with parasite infection in the eye needs to be further explored.

NK cells and CD49a^+^ NK subsets use surface receptors (activating, inhibitory and cytokines) to investigate damage or infection of host cells and tissues. Receptor engagement stimulates the killing of diseased target cells (cytotoxicity) and initiates the production of IFN-γ, which may eliminate most *T*. *gondii*. NK cells may alter immunologic balance in immune diseases by regulating the secretion of cytokines or by interacting with other cells through their surface receptors [38, 50–55]. Accordingly, regulation of NK-cellular function could prove beneficial in contributing to the treatment of immune diseases. Many factors are involved in mediating the function of NK cells. For example, the expression of surface molecules on NK cells, including inhibitory signals, activating signals, and adhesion molecules, can all be important factors in determining NK-cell function [56].Our *in vivo* study validates the upregulation of ICAM-1 expression in ocular tissue following *T*. *gondii* infection, which is consistent with the findings of Nagineni *in vitro* [20].More importantly, we discovered that the expression of its receptor LFA-1 was also upregulated both on NK cells and CD49a^+^ NK cells, which has not yet been reported. Using a murine model of intraocular *T*. *gondii* infection, this study revealed for the first time that the overexpression of LFA-1/ICAM-1 may be involved in the immunopathology process of OT.

Preclinical studies on various ocular diseases have shown that inhibition of the interaction between integrins and their ligands, particularly LFA-1 and ICAM-1, holds promise as a therapeutic approach. Anti-LFA-1 monoclonal antibody was approved for the treatment of psoriasis but was withdrawn from the market because of its side effects. Lifitegrast, by virtue of its high efficacy and low local and systemic toxicity, is the optimal drug for ocular use in dry eye disease (DED) patients [35, 57, 58]. Lifitegrast ophthalmic drops administered thrice daily provide therapeutic levels of lifitegrast in the retina and can reduce retinal complications associated with diabetes [59]. In murine endotoxin-induced uveitis, an acute inflammatory model, a decreased number of infiltrating leukocytes was observed in animals receiving either LFA-1- or ICAM-1-neutralizing antibodies [60]. Interestingly, although the percentage of total NK-cell and CD49a^+^ NK-cell in the Lifitegrast 2 group (from Day 14) did not change, the percentage of CD49a^+^ NK-cell in the Lifitegrast 1 group (from Day 0) was significantly decreased after lifitegrast treatment, possibly suggesting that lifitegrast may inhibit the ocular inflammatory response by reducing CD49a^+^ NK-cell subsets during the early process. As other immune cells also use LFA-1/ICAM-1 adhesion receptors, lifitegrast could also have the potential to improve the pathology of OT by affecting the status of other immune cells, such as T cells or DCs. The exact effects of LFA-1/ICAM-1 on lymphocytes in OT will require additional investigation. Lifitegrast not only altered the percentage of CD49a^+^ NK cells in the eyes but also in the spleen, suggesting that lifitegrast affected inflammatory cells in the eyes as well as the systemic immune system. We examined the effect of lifitegrast on LFA-1 and ICAM-1 expression and found that it significantly inhibited LFA-1 and ICAM-1 protein expression in lifitegrast-treated groups. In addition, it significantly inhibited LFA-1 expression on NK cells in Lifitegrast 1 group (from Day 0) but not Lifitegrast 2 group (from Day 14). We speculate that this may be related to the fact that the immune response of NK cells might have occurred in the early process of infection. Lifitegrast is a = novel small molecule integrin antagonist. Based on the current understanding of its mechanism of action, lifitegrast could block the recruitment and activation of T cells to the ocular surface [61]. Signal transduction mediated by LFA-1 and ICAM-1 is involved in the adhesion, migration, and activation processes of lymphocytes [18]. In this study, we found that the percentage of CD49a^+^ NK cells in the eye was decreased after lifitegrast treatment. Therefore, we speculated that the inhibition of LFA-1 expression may be associated with a decrease in the immune cell percentage. Furthermore, the inhibition of NK-cell activation during infection may also suppress LFA-1 (activating receptor) expression [62].

Our results found that lifitegrast treatment ameliorated the pathology of OT even in the Lifitegrast 2 group, and may open a new phase for potential immunotherapy. Among the most prominent cytokines secreted by NK cells are IFN-γ and TNF-α. Moreover, NK cells have been reported to produce several other immunoregulatory cytokines, including IL-10, IL-5, and IL-8 [63].The downstream effect of lifitegrast on cytokines has been reported in multiple/several preclinical studies [64]. Both cytokine production in the retina and the whole eye was shown in this study, and changes in cytokines in the retina followed the same trend as those in the whole eye. *T*. *gondii* can infect the whole eye, including the cornea, iris/ciliary body, lens, peripheral retina, posterior retina, sclera, and choroid [27]. In this study, we detected NK cells throughout the whole eye, and the pathological effects they mediate may occur not only in the retina but also in other ocular tissues. The retinal endothelium is the primary target of *T*. *gondii* in OT because of the high constitutive expression of ICAM-1 transcripts by human retinal endothelial cells [22, 65, 66]. We focused on observing the histopathology of the retina in our study. In addition, the role of NK cells in other ocular tissues requires further study.

Activation of LFA-1 receptors can be divided into two signaling processes: those responsible for cell proliferation or those preventing apoptosis [67]. Caspase-3 is a key enzyme in the execution of apoptosis [68]. Our study explores the possible mechanisms underlying the reduction in infection in OT that affect cytokines and inhibit apoptosis. Further studies are needed to infer any additional roles that LFA-1/ICAM-1 may play in the treatment of OT and its regulatory mechanism in NK cells.

In conclusion, our study revealed for the first time the increased percentage of NK cells, specifically CD49^+^ NK cells, upon infection and overexpression of LFA-1 in NK cells and ICAM-1 in an OT murine model. Blocking the binding of LFA-1 to ICAM-1 with lifitegrast, decreased the CD49a^+^ NK-cell proportion during early treatment and reduced the severity of OT, which is associated with reduced LFA-1/ICAM-1 expression in the eyes and may provide a new target for treatment.

## Supporting information

S1 Table Regulatory genes in KEGG enrichment. (XLSX)
